# Long non-coding RNA PXN-AS1 suppresses pancreatic cancer progression by acting as a competing endogenous RNA of miR-3064 to upregulate PIP4K2B expression

**DOI:** 10.1186/s13046-019-1379-5

**Published:** 2019-09-05

**Authors:** Jiayan Yan, Yunxi Jia, Han Chen, Wei Chen, Xiaoying Zhou

**Affiliations:** 1 Department of Biliary-Pancreatic Surgery, Renji Hospital, School of Medicine, Shanghai Jiaotong University, Shanghai, 200127 China; 2 Department of endoscopy of geriatric gastroenterology, First Affiliated Hospital of Nanjing Medical University, Nanjing, 210029 China; 3 Department of gastroenterology, First Affiliated Hospital of Nanjing Medical University, Nanjing, 210029 China

**Keywords:** miR-3064, PXN-AS1, PIP4K2B, Pancreatic cancer

## Abstract

**Background:**

Dysregulation of microRNAs (miRNAs) play critical roles in cancerous processes. Although miR-3064 was reported to be an important tumor suppressor in ovarian cancer, the cellular impact of miR-3064 on pancreatic cancer (PC) progression, its downstream target genes and upstream mechanisms that control the expression of miR-3064 remain to be fully clarified.

**Methods:**

We compared miRNA expression profiles between PC tissues compared with normal tissues using a miRNA microarray analysis of clinical samples, and screened the identified miRNAs for their influence on cell proliferation. We measured the expression of miR-3064 in PC tissues and PC cell lines using quantitative real-time PCR assays. Gain- and loss-of-function experiments were conducted to explore the biologic significance of miR-3064 in PC progression both in vitro and in vivo. The interactions between miR-3064 and long noncoding RNA (lncRNA) PXN-AS1 was verified using the luciferase reporter assay and RNA immunoprecipitation assay.

**Results:**

We showed that miR-3064 was significantly overexpressed in PC tissues compared to normal tissues. High miR-3064 was associated with worse prognosis in patients with PC. Functionally, ectopic expression of miR-3064 promoted the proliferation, invasion, clone formation and sphere formation of PC cells in vitro and stimulated PC growth in vivo, while specific knockdown of miR-3064 or CRISPR/Cas9-mediated knockout of miR-3064 resulted in opposite phenotypes. Further investigation revealed that miR-3064 directly targeted PIP4K2B, which was reduced in PC tissues and attenuated PC cell proliferation, invasion and sphere formation induced by miR-3064. Importantly, lncRNA PXN-AS1 expression was downregulated in PC samples, and it directly interacted with miR-3064 and suppressed its levels in PC cells. Enforced expression of PXN-AS1 remarkably decreased cell proliferation, invasion and sphere formation, while re-expression of miR-3064 abrogated these effects of PXN-AS1.

**Conclusions:**

MiR-3064, a key oncogenic miRNA, could promote PC cell growth, invasion and sphere formation via downregulating the levels of tumor suppressor PIP4K2B. PXN-AS1 functioned as a sponge to suppress the expression of miR-3064. These observations offer fresh insight into the mechanisms through which miR-3064 modulates the development of PC.

**Electronic supplementary material:**

The online version of this article (10.1186/s13046-019-1379-5) contains supplementary material, which is available to authorized users.

## Background

Pancreatic cancer (PC) is considered the most fatal gastrointestinal malignancy worldwide [1]. In most case, PC is usually clinically silent at the early stage, with symptoms only developing at an advanced stage [1]. Therapeutic options remain very limited even if some progress has been achieved in the development of combination therapies [2]. Therefore, further explorations of the mechanisms of PC progression are urgently needed and will provide new opportunities to develop effective therapeutic strategies against PC.

Recent genome-wide studies have identified thousands of long noncoding RNAs (lncRNAs), which are RNAs longer than 200 nucleotides without protein-coding potential [3, 4]. Increasing evidence has shown that lncRNAs are differentially expressed and act as potent regulators of tumor progression and metastasis in various cancers including PC [4]. LncRNAs regulate gene expression through several mechanisms, such as transcriptional regulation, chromatin remodeling, histone modification, regulation of mRNA splicing and stability and acting as sponges for microRNAs (miRNAs) [5–7].

MiRNAs are small, noncoding RNAs that inhibit protein translation and/or negatively regulate mRNA stability by binding to the 3′-untranslated region (3′-UTR) of target mRNAs [8]. Depending on the functions of their target mRNAs, miRNAs can act as both tumor oncogenes and tumor suppressors [9, 10]. A previous study has reported that the expression levels of miR-3064 was downregulated in ovarian cancer samples, and can inhibit the proliferation, epithelial-mesenchymal transition and invasion of ovarian cancer cells [11], but the precise cellular roles, mechanisms by which miR-3064 influences PC progression, and the molecular events that modulate the levels of miR-3064 in PC, remains largely unknown.

In this study, we focused on miR-3064 and explored its downstream and upstream signaling pathway during PC progression. We found that miR-3164 acts as an oncogenic miRNA by targeting tumor suppressor PIP4K2B in PC, and lncRNA PXN-AS1 could reduce the expression of miR-3064 by functioning as a sponge for miR-3064. Collectively, our data suggest that this PXN-AS1/miR-3064/PIP4K2B pathway has the potential for being used in diagnosis and treatment for PC.

## Materials and methods

### Collection of specimens

This study was approved by the Ethics Committee of Renji Hospital, School of Medicine, Shanghai Jiaotong University. Each patient signed the written informed consent before enrollment in the study. Human PC specimens and adjacent noncancerous tissues were collected from 50 patients with PC who were treated at the Department of Biliary-Pancreatic Surgery, Renji Hospital, School of Medicine, Shanghai Jiaotong University between 2010 and 2015 (Tables 1, 2 and 3). No patients received chemotherapy or radiotherapy prior to surgery. All the specimens were evaluated by two pathologists in a double-blind manner. The specimens were immediately snap-frozen in liquid nitrogen and stored at − 80 °C before use.

### Cell lines, vectors, siRNAs and transfection

Five PC cell lines (AsPC-1, BxPC-1, PANC-1, SW1990 and PaCa-2) and a normal human pancreatic duct epithelial cell line HPDE6-C7 were purchased from the American Type Culture Collection (Rockville, USA), and were cultured in RPMI 1640 (Invitrogen, Shanghai, China), supplemented with 10% fetal bovine serum (FBS). All cells were cultured at 37 °C with 5% CO_2_.

The vectors encoding lncRNA *PXN-AS1* or *PIP4K2B*, the control vector, PXN-AS1 siRNAs, PIP4K2B siRNAs, control siRNA, miRNA mimic, control mimic, miRNA inhibitors and control inhibitor were purchased from IGEbio (Guangzhou, China). Cell infection was performed using Lipofectamine 2000 (Invitrogen, Carlsbad, CA, USA) following the manufacturer’s protocol.

### RNA extraction, miRNA microarray and qRT-PCR assay

Total RNA was extracted from tissues or PC cells using TRIzol reagent (Invitrogen, Grand Island, NY, USA) according to the manufacturer’s instructions. The quantity and quality of the RNA were determined using a NanoPhotometer spectrometer (ImplenInc, CA, USA). MicroRNA expression profiles of PC specimens and adjacent noncancerous tissues (*n* = 5 per group) were analyzed using the Affymetrix Genechip miRNA 4.0 array (KangChen Biotech, Shanghai, China) according to the manufacturer’s instructions. Data were obtained and analyzed with AGCC software (Affymetrix, Santa Clara, CA, USA). Fold change greater or equal to 2 have been considered as significant change.

Total RNA (500 ng) was reverse transcribed to get the first-strand cDNA using the Prime Script RT Reagent Kit (Takara, Dalian, China). Quantitative PCR assays were carried out on an ABI 7500 real-time PCR system (Applied Biosystems, Foster City, CA, USA) using the SYBR Premix Ex Taq (Takara, Dalian, China). Results were normalized to the expression of GAPDH. The mirVanaTM qRT-PCR microRNA Detection Kit (Ambion Inc., Austin, TX, USA) was used to assess the levels of miR-3064 according to the manufacturer’s instructions. U6 was used for data normalization.

### Western blot analysis and antibodies

An equal amount of cell protein lysates were separated by 10% sodium dodecyl sulfate (SDS) polyacrylamide gel electrophoresis, transferred to polyvinylidene fluoride membranes (Millipore, Billerica, MA, USA), and incubated with specific antibodies, PIP4K2B (1:2000, Santa Cruz, CA, USA) and GAPDH (1:5000, Santa Cruz, CA, USA). Bands were visualized with ECL detection reagents (Amersham Biosciences, Buckinghamshire, UK). GAPDH was used as a control.

### Cell proliferation assay

Cell proliferation assays were performed using the CCK-8 assay (Beyotime Institute of Biotechnology, Jiangsu, China) according to the manufacturer’s instructions. Briefly, the cells were seeded into the 96-well plates at a density of 5000 cells per well. 10 μl of the CCK-8 solution was added to each well of cells. After 1 h of incubation, the absorbance of each well was measured using a microplate reader (Bio-Rad, Hercules, CA, USA).

### Cell invasion assay

Cell invasion assays were conducted as reported previously [12]. Fifty thousand cells were added into the upper chambers of the Matrigel-coated transwell plates (Corning Costar Co, Lowell, CA, USA). 750 μl of serum-containing medium was added to the lower chambers. After 24 h of incubation, the cells that have invaded were fixed with 4% paraformaldehyde for 30 min, washed 3 times with PBS (Gibco, Grand Island, NY, USA), and stained with 0.1% crystal violet for 15 min (Beyotime, Shanghai, China). The cells were counted under a microscope, and ten random fields of view were analyzed for each chamber.

### Colony formation assay

PC cells were plated into 6-well plates (500 cells per well) and cultured for 2 weeks. Colonies were fixed and stained with 0.4% crystal violet (Bio Basic Inc., Markham, Canada) in 20% ethanol for 5 min. Cell colonies were photographed and counted.

### Sphere formation assay

PC cells were digested, counted and seeded in 6-well ultra-low attachment plates (Corning Incorporated, Corning, NY, USA) at a density of 5000 cells/well in serum-free RPMI 1640 medium (Invitrogen, Shanghai, China) containing 20 ng/ml human FGF (Gibco, Thermo Fisher Scientific, Waltham, MA, USA), 20 ng/ml human EGF (Gibco), 1% N2 supplement (Gibco) and 1% B27 (Gibco). Cells were cultured at 37 °C in an atmosphere containing 5% CO2 for 2 weeks to form spheres. After 2 weeks, the number of spheres was counted and recorded under a light microscope (Nikon Corporation).

### CRISPR-Cas9-mediated knockout of *miR-3064*

In order to efficiently target *miR-3064* for knockout, CRISPR guide RNA (gRNA) sequences (gRNA-1: TATTTTTGGTCTGGCTGTTG; gRNA-2: CTAACTTTTTATTTTTGGTC) were selected from the human GeCKOv2 CRISPR knockout pooled library [13], which contains CRISPR gRNAs that target every gene and miRNA in the genome. Then, these gRNAs were cloned into pLenti-CRISPR V2 vector (Addgene plasmid #52961, which expresses a single gRNA under the U6 promoter and the wild type Cas9 nuclease under the EFS promoter). The lenti-CRISPR-control vector expressing a gRNA targeting *EGFP* was constructed by inserting a gRNA sequence targeting human *EGFP* sequence into the pLenti-CRISPR V2 vector lentiviral vector as previously reported [14].

Lentiviral particles encoding gRNAs targeting *miR-3064* gene or a control gRNA sequence targeting *EGFP* gene were produced in human HEK293FT cell line (Invitrogen, Carlsbad, CA, USA) using Virapower Lentiviral Expression Kit (Invitrogen, Carlsbad, CA, USA) according to the manufacturer’s instructions. The medium was changed after 6 h of incubation at 37 °C and 5% CO_2_. The first and second viral supernatants were collected 24 and 52 h after transfection, respectively. Harvested viral supernatants were filtered through a 0.22 μm membrane and stored at − 80 °C.

To evaluate the effect of targeting *miR-3064* by gRNAs, PaCa-2 cells were transduced with the harvested lentiviral particles as indicated. Briefly, approximately 2 × 10^4^ cells were seeded in a 24-well plate. PaCa-2 cells were then transduced in the presence of 8 μg/ml of polybrene (Sigma-Aldrich, St Louis, MO, USA) with lentiviral particles. Approximately 48 h post-infection, the cells were selected by treating with 5 μg/ml of Puromycin (Sigma-Aldrich, St Louis, MO, USA) for 7 days.

The resulting cells were clonally expanded by isolating single cells using a limiting dilution approach. Next, single cell clones were picked up and cultured in 96-well plates. After 7 days, the cell colonies were sequentially subcultured in 24- and 6-well plates with 2.5 μg/ml of Puromycin for another 10 days. Subsequently, a fraction of selected cells were subjected to sequencing analysis.

To determine the mutation, genomic DNA was extracted using a PureLink Genomic DNA Mini Kit (Invitrogen, Carlsbad, CA, USA) and regions surrounding gRNA target sites within the *miR-3064* gene were amplified by PCR using Amplitaq Gold 360 PCR Master Mix (Invitrogen, Shanghai, China). PCR reactions were purified using a GeneJET PCR Purification Kit (Thermo Scientific, Waltham, MA, USA). Amplicons were then analyzed by Sanger sequencing (KangChen Biotech, Shanghai, China).

### Xenograft assay

All animal procedures were approved by the Institutional Animal Care and Use Committee of Renji Hospital, School of Medicine, Shanghai Jiaotong University. All the methods were conducted in conformity with the relevant guidelines and regulations about animals and humans. BALB/c nude mice (4 weeks old) were obtained from Beijing HFK Bioscience (Beijing, China) and maintained under pathogen-free conditions. PC cells were injected into subcutaneously in the right flank of the nude mice. The tumor volumes and weights were measured every 3 days in the mice; the tumor volumes were measured as length × width^2^ × 0.5. 3 weeks after injection, the mice were killed, and the tumors were collected for further analysis. The Ki-67 levels were determined with immunohistochemistry assay. The primary anti-human Ki-67 antibody (1:1000, Abcam, Cambridge, UK) was incubated with tissues at 4 °C overnight. On the next day, the tissues were washed and incubated with biotin-labeled rabbit anti-mouse IgG (1:200; Sigma-Aldrich, St Louis, MO, USA). 3, 3′-Diaminobenzidine (ab64238, Abcam, Cambridge, UK) was used to stain the tissues.

### Dual-luciferase reporter gene assay

The reporters containing wild-type (WT) *PXN-AS1*, mutated (MUT) *PXN-AS1* with the mutated miR-3064 binding site, or WT *PIP4K2B* 3′-untranslated region (3′-UTR), or MUT *PIP4K2B* 3′-UTR with the mutated miR-3064 binding site, were obtained from IGEbio (Guangzhou, China). Mutations of the *PXN-AS1* fragment or *PIP4K2B* 3′-UTR in the luciferase reporter construct was generated by PCR mutagenesis using a QuickChange site-directed mutagenesis kit (Stratagene, La Jolla, CA, USA) according to the manufacturer’s directions.

Cells were seeded at a density of 2 × 10^5^ cells/well in 24-well plates and co-transfected after 24 h with 0.2 μg of reporter plasmid, 0.002 μg of Renilla luciferase internal control plasmid (pRL-CMV, Promega, Madison, WI, USA), as well as 50 nM of miR-3064 mimic, 50 nM of miR-3064 inhibitor, or the respective negative controls per well using Lipofectamine 2000 (Invitrogen, Carlsbad, CA, USA). At 48 h after transfection, the relative luciferase activity was confirmed following the Dual-Luciferase Reporter Assay Kit instructions (Promega, Madison, WI, USA).

### RNA immunoprecipitation (RIP) assay

RIP assays were conducted using the Magna RIP RNA-Binding Protein Immunoprecipitation Kit (Millipore, Bedford, MA, USA). PC cells were lysed in the RIP-lysis buffer. Then, 100 μl of whole-cell extracts were incubated with magnetic beads conjugated with the human anti-Ago2 antibody (Millipore, Bedford, MA, USA) or normal mouse IgG (Millipore, Bedford, MA, USA) overnight at 4 °C. The samples were then incubated with Proteinase K to digest the proteins, and finally immunoprecipitated RNA was isolated with TRIzol reagent (Invitrogen, Grand Island, NY, USA), and was used for qRT-PCR analysis.

### Statistical analysis

Data are presented as mean ± standard deviation. Statistical analysis was performed using the SPSS 19.0 statistical software (SPSS, Chicago, USA). The Student’s *t*-test, Chi-Squared test, ANOVA and Wilcoxon signed-rank test were used to analyze the significance of difference between groups. A value of *p* < 0.05 was considered as statistically significant.

## Results

### MiR-3064 is highly expressed in PC cell lines and tissues

**Fig. 1:**
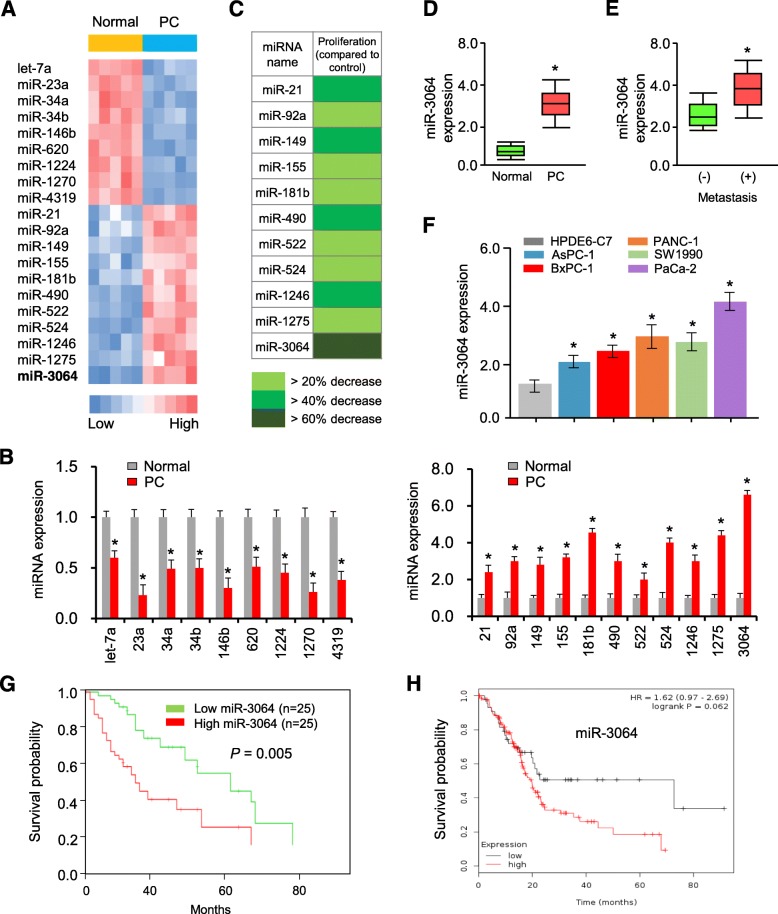
Identification of miR-3064 as a potential oncogenic miRNA in PC. (**a**) Heatmap of miRNAs expression in human PC tissues (*n* = 5) compared with normal tissues (*n* = 5) measured by miRNA microarray. (**b**) qRT-PCR analysis of indicated miRNAs in PC tissues (*n* = 5) and normal tissues (*n* = 5). (**c**) Effects of transfection with 11 upregulated miRNAs on PC cell proliferation. (D) Expression of miR-3064 in PC tissues and normal tissues using qRT-PCR analysis (*n* = 50). (**e**) Levels of miR-3064 in PCs with lymph node metastasis tissues and PCs without node metastasis. (**f**) The expression of miR-3064 was examined in five PC cell lines and a normal pancreatic cell line HPDE6-C7 using qRT-PCR analysis. (**g**) Kaplan-Meier curves for overall survival of PC patients with low or high miR-3064 levels. (**h**) Kaplan–Meier analysis for overall survival based on low or high miR-3064 levels (the KMPlotter database). *P < 0.05

First, we performed a miRNA microarray analysis to evaluate miRNA expression profiles in human PC specimens and adjacent noncancerous tissues. Microarray expression profiling revealed 20 miRNAs (11 up-regulated and 9 down-regulated) that were differentially expressed in PC specimens compared with normal tissues (Fig. 1a). For validation, qRT-PCR analysis for these 20 miRNAs was carried out in fresh tissues (50 cases of PC and 50 cases of normal tissues). The results of qRT-PCR assays confirmed the miRNA microarray results whereby these miRNAs were differentially expressed in PC samples compared with normal samples (Fig. 1b). Among the identified miRNAs, serval (including let-7a, miR-34a, miR-100 and miR-124) acts as known tumor suppressors, and some (such as miR-21, miR-92a and miR-181) are known to be oncogenic miRNAs in PC [15]. Because the expression of miR-3064 was most increased in PC samples (Fig. 1b), it was considered as an important miRNA for PC formation and progression.

Next, we hypothesized that the upregulated miRNAs may contribute to increased PC cell proliferation. To test this hypothesis, we transfected the PC cell line PANC-1 with miRNA inhibitors for each of the 11 miRNAs and investigated the impacts of these miRNAs on cell proliferation. Although the inhibition of all these miRNAs suppressed cell proliferation, miR-3064 attenuated cell growth to a greater extent than the remaining miRNAs (Fig. 1c). The overexpression of miR-3064 was subsequently validated in a cohort of 50 PC patients (Fig. 1d).

Moreover, when 50 samples from PC patients were classified based on the occurrence of lymph node metastasis, we found that the expression levels of miR-3064 were significantly higher in PCs with lymph node metastasis compared with those without metastasis (Fig. 1e). To further address the correlation between miR-3064 levels and the clinicopathological characteristics of PC patients, we classified 50 PC patients into two groups: the high miR-3064 group and the low miR-3064 group based on the median value of miR-3064 expression. We found that higher miR-3064 expression was significantly associated with increased tumor size, advanced TNM stage and the presence of lymph node metastasis (Table 1). The levels of miR-3064 in five PC cell lines were significantly up-regulated compared to HPDE6-C7, a normal human pancreatic cell line (Fig. 1f).

**Table 1:** Correlations between miR-3064 expression and clinicopathological characteristics of PC patients

Characteristics	miR-3064 expression		*P*-value
	Low	High	
Age (years)			
> 60	13	14	0.349
≤ 60	13	10	
Differentiation			
Well/moderate	12	14	0.749
Poor	14	10	
Tumor size			
≤ 2 cm	15	3	**0.01**
> 2 cm	11	21	
TNM Stage			
I/II	17	4	**0.005**
III/IV	9	20	
Lymph node metastasis			
Positive	9	21	**0.002**
Negative	17	3	

*Statistically significant (*P* < 0.05)

We investigated the potential clinical significance of miR-3064 in PC. Follow-up data were available for all the 50 patients included in this study. We found that those patients with higher miR-3064 expression showed a significantly shorter overall survival (Fig. 1g). To further explore the prognostic significance of miR-3064 expression in PC patients from the TGCA dataset, we used an open online database KMPlotter [16], where a total of 178 patients were classified into two groups (high-expression and low-expression groups) according to the median level of miR-3064 expression in cancer tissues. As a result, the patients with higher miR-3064 expression had worse overall survival than those patients with lower miR-3064 expression (Fig. 1h), suggesting an oncogenic role for miR-3064 in mediating the malignant features of PC cells.

Therefore, we focused our efforts on the role of miR-3064 in the tumorigenesis and progression of PC, and the identification of its upstream regulators and downstream target genes.

### MiR-3064 promotes the aggressive oncogenic phenotype of PC cells in vitro and tumor growth in vivo

**Fig. 2:**
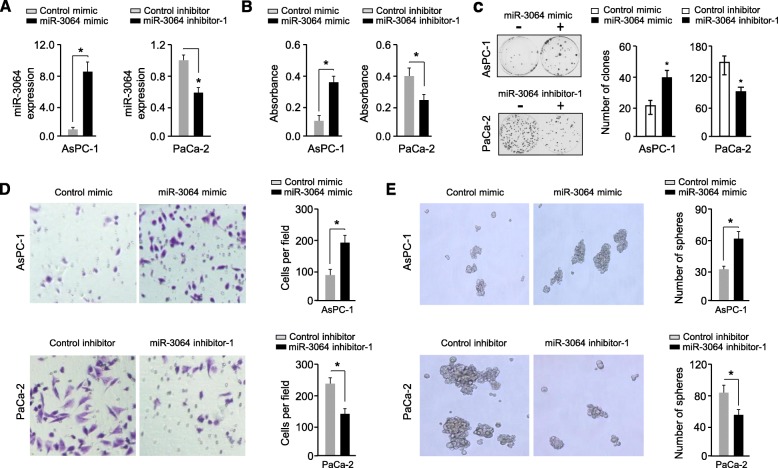
MiR-3064 increases the proliferation, clone formation, invasion and sphere formation of PC cells in vitro*.* (**a**) Verification of miR-3064 overexpression or knockdown in PC cells using qRT-PCR analysis. (**b**-**e**) CCK-8 assay (**b**), clone formation assays (**c**), cell invasion assays (**d**) and sphere formation assays (**e**) in AsPC-1 cells after overexpression of miR-3064 and in PaCa-2 cells after knockdown of miR-3064. *P < 0.05

Given that miR-3064 is involved in the regulation of multiple cellular functions associated with tumor malignancy [11], we functionally analyzed the phenotypic consequences of a modified miR-3064 expression in PC cells. AsPC-1 cells endogenously express lower levels of miR-3064, while another PC cell line PaCa-2 expresses high miR-3064 levels (Fig. 1e). Hence, we transiently transfected AsPC-1 cells with miR-3064 mimic, and knocked down miR-3064 in PaCa-2 cells with miR-3064 inhibitors (Fig. 2a and Additional file 1: Figure S1A).

CCK-8, clone formation, invasion and sphere formation assays showed that enforced miR-3064 expression significantly enhanced cellular proliferation, clone formation, invasion and sphere formation in ASPC-1 cells (Fig. 2b-e). However, miR-3064 knockdown significantly suppressed the proliferation, clone formation, invasion and sphere formation in PaCa-2 cells (Fig. 2b-e and Additional file 1: Figure S1B-E). These observations suggested that upregulation of miR-3064 promotes the aggressiveness of PC cells in vitro.

**Fig. 3:**
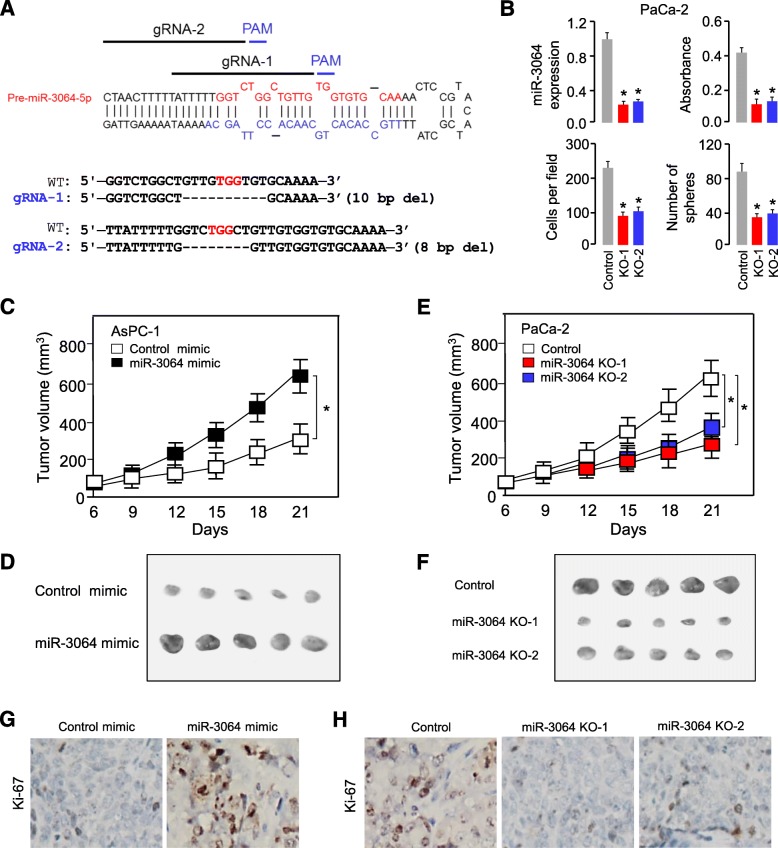
Depletion of miR-3064 suppresses the aggressive phenotypes of PC cells in vitro and inhibits tumor growth in xenograft mouse models. (**a**) Schematic diagram of gRNAs targeting at *miR-3064* locus (upper). DNA sequencing confirmed the deletions generated by CRISPR/Cas9 system in the *miR-3064* locus (bottom). (**b**) CRISPR/Cas9 with designed gRNAs significantly reduced the expression of miR-3064, and suppressed the proliferation, invasion and sphere formation of PaCa-2 cells. (**c**, **d**) AsPC-1 cells were transfected with or without miR-3064 mimic, and injected into nude mice. Tumor growth rates (**c**) and images (**d**) of xenograft tumors were shown. (**e**, **f**) The miR-3064 knockout or control PaCa-2 cells were injected into nude mice, and tumor growth rate (**e**) and images (**f**) of xenograft tumors were shown. (**g**, **h**) Immunohistochemical staining of Ki-67 in tumors derived from (**c** and **e**). *P < 0.05

We noticed that only a moderate decrease in the expression of miR-3064 was achieved by the transfection with miR-3064 inhibitor (Fig. 2a). To determine if the use of the CRISPR/Cas9 system targeting *miR-3064* genomic DNA loci could robustly suppress miR-3064 expression in PaCa-2 cells, we constructed CRISPR/Cas9 vectors containing gRNAs with complementary sequences to *miR-3064* (Fig. 3a). Genomic DNA was isolated from several clones and then sequenced. We confirmed the CRISPR/Cas9-induced deletions in the *miR-3064* locus (Fig. 3a). Our qRT-PCR analysis demonstrated that the expression levels of miR-3064 were dramatically reduced in PaCa-2 cells transduced with the lenti-CRISPR-miR-3064 vectors when compared to control cells transduced with the control vector (Fig. 3b). Knockout of miR-3064 by CRISPR/Cas9 markedly suppressed the proliferation, invasion and sphere formation ability of PaCa-2 cells (Fig. 3b).

Moreover, we assessed the effects of ectopic miR-3064 expression on PC formation in vivo. Enforced miR-3064 expression in AsPC-1 cells resulted in increased growth and tumor size of subcutaneous xenograft tumors in nude mice (Fig. 3c and d). We also investigated whether miR-3064 knockout could regulate tumor growth in vivo. PaCa-2 cells transfected with the lenti-CRISPR-miR-3064 vectors or control vector were subcutaneously injected into nude mice. Knockout of miR-3064 strikingly inhibited PaCa-2 xenograft tumor growth in mice, as determined by tumor growth rates and tumor size (Fig. 3e and f). Furthermore, immunohistochemical staining studies demonstrated that the tumors originating from miR-3064-overexpressing AsPC-1 cells had increased expression of Ki-67, compared with that from the control cells (Fig. 3g). In contrast, the tumors formed from the miR-3064 knockout PaCa-2 cells had a decreased expression of Ki-67 (Fig. 3h). Thus, these results showed that miR-3064 can promote the aggressive phenotypes of PC cells in vitro and display tumor-promoting activity in vivo.

### MiR-3064 targets PIP4K2B to repress its expression in PC cells

**Fig. 4:**
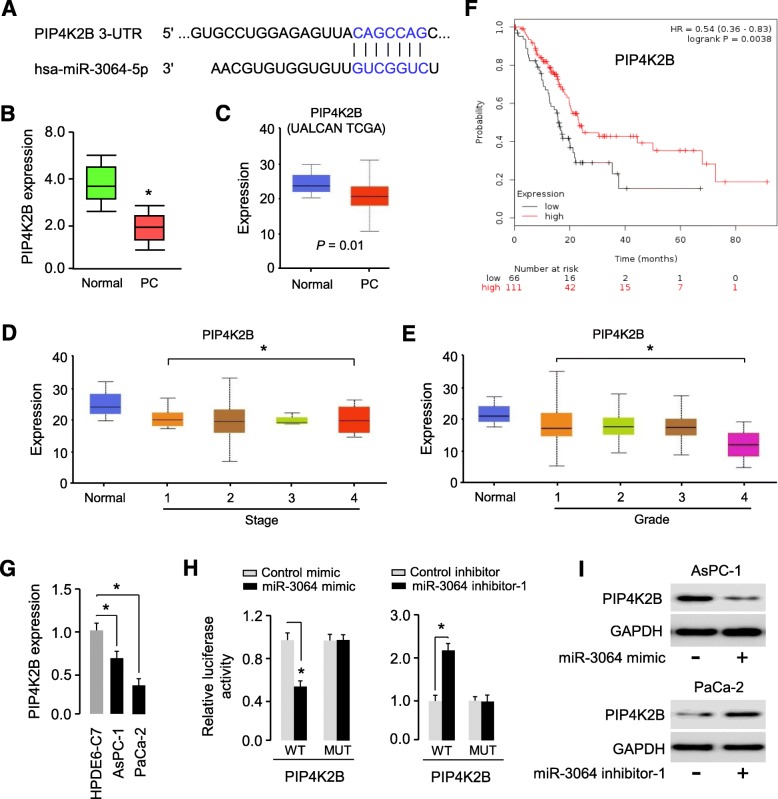
MiR-3064 targets PIP4K2B and represses its expression in PC cells. (**a**) The predicted binding sites between miR-3064 and *PIP4K2B* 3′-UTR. (**b**) qRT-PCR analysis of miR-3064 expression in PC tissues and normal tissues. (**c**) Analysis of PIP4K2B expression in PC tissues and normal tissues using the TCGA data obtained from the UALCAN database. (**d**, **e**) miR-3064 expression in PCs classified by tumor stages (**d**) and grades of differentiation (**e**) according to UALCAN database. (**f**) Association of PIP4K2B expression with overall survival in PC patients using the KMplotter database. (**g**) Examination of PIP4K2B expression in PC cell lines and a normal pancreatic cell line HPDE6-C7 using qRT-PCR assays. (**h**) The luciferase activity in AsPC-1 cells cotransfected with reporter vectors containing wild type (WT) or mutant (MUT) *PIP4K2B* 3′-UTR, together with or without miR-3064 mimic, and in PaCa-2 cells cotransfected with reporter vectors containing wild type or mutant *PIP4K2B* 3′-UTR, together with or without miR-3064 inhibitor. (**i**) The protein levels of PIP4K2B in AsPC-1 cells transfected with or without miR-3064 mimic, and in PaCa-2 cells transfected with or without miR-3064 inhibitor. *P < 0.05

By using the TargetScan algorithm, we identified PIP4K2B as a candidate target for miR-3064. The seed region of miR-3064 could form complementary base pairs with the 3′-UTR of *PIP4K2B* mRNA (Fig. 4a). The qRT-PCR results suggested that PC tissues expressed a lower level of PIP4K2B than the adjacent normal tissues (Fig. 4b). By analyzing the expressions of PIP4K2B in PC tissues and normal tissues using the TCGA data obtained from the UALCAN database (http://ualcan.path.uab.edu/), we found that PIP4K2B levels were reduced in cancer tissues compared with normal tissues (Fig. 4c).

We also analyzed the expression of PIP4K2B in PC samples classified by histological pathological stage and tumor grade using the UALCAN database. The levels of PIP4K2B in PC tissues decreased gradually from stages I to IV (Fig. 4d). When PCs were categorized by tumor grades, PIP4K2B expression was progressively downregulated in tumors with advanced grades (Fig. 4e). We observed that increased tumor size, advanced TNM stage and the presence of lymph node metastasis were associated with reduced PIP4K2B expression in PC patients (Table 2).

**Table 2:** Correlations between PIP4K2B expression and clinicopathological characteristics of PC patients

Characteristics	PIP4K2B expression		*P*-value
	Low	High	
Age (years)			
> 60	14	13	0.776
≤ 60	11	12	
Differentiation			
Well/moderate	15	11	0.785
Poor	10	14	
Tumor size			
≤ 2 cm	3	15	**0.00007**
> 2 cm	26	6	
TNM Stage			
I/II	3	18	**0.0001**
III/IV	22	7	
Lymph node metastasis			
Positive	23	7	**0.00001**
Negative	2	18	

*Statistically significant (*P* < 0.05)

To further evaluate whether PIP4K2B levels were associated with the prognosis of PC patients, we carried out a survival analysis of PC patients using the KMPlotter database. We found that patients with higher PIP4K2B expression levels had better overall survival than those with lower PIP4K2B expression levels (Fig. 4f). Meanwhile, qRT-PCR assays established that PIP4K2B expression was reduced in PC cell lines (AsPC-1 and PaCa-2 cells), compared with a normal cell line HPDE6-C7 (Fig. 4g). All these results indicated a negative association between miR-3064 and PIP4K2B expression in PC cells.

To verify whether miR-3064 could bind to the 3′-UTR of *PIP4K2B*, we performed the luciferase reporter assays. The luciferase activity was significantly decreased in miR-3064 mimic-transfected cells, whereas was induced in miR-3064 inhibitor-transfected cells (Fig. 4h). Furthermore, the point mutations of the miR-3064 targeting site were able to abolish the overserved effects of miR-3064 on the 3′-UTR of *PIP4K2B* (Fig. 4h). To ask whether the protein levels of PIP4K2B was regulated by miR-3064, we used miR-3064 mimics or miR-3064 inhibitor to change the endogenous miR-3064 levels and conducted western blot analysis. PIP4K2B protein expression was reduced after the transfection with miR-3064 mimic, whereas PIP4K2B expression was induced after the transfection with miR-3064 inhibitor (Fig. 4i). These results suggested that miR-3064 represses PIP4K2B expression through the predicted targeting site in the *PIP4K2B* 3′-UTR.

### Restoration of PIP4K2B reverses the tumor-promoting roles of miR-3064 in PC cells

**Fig. 5:**
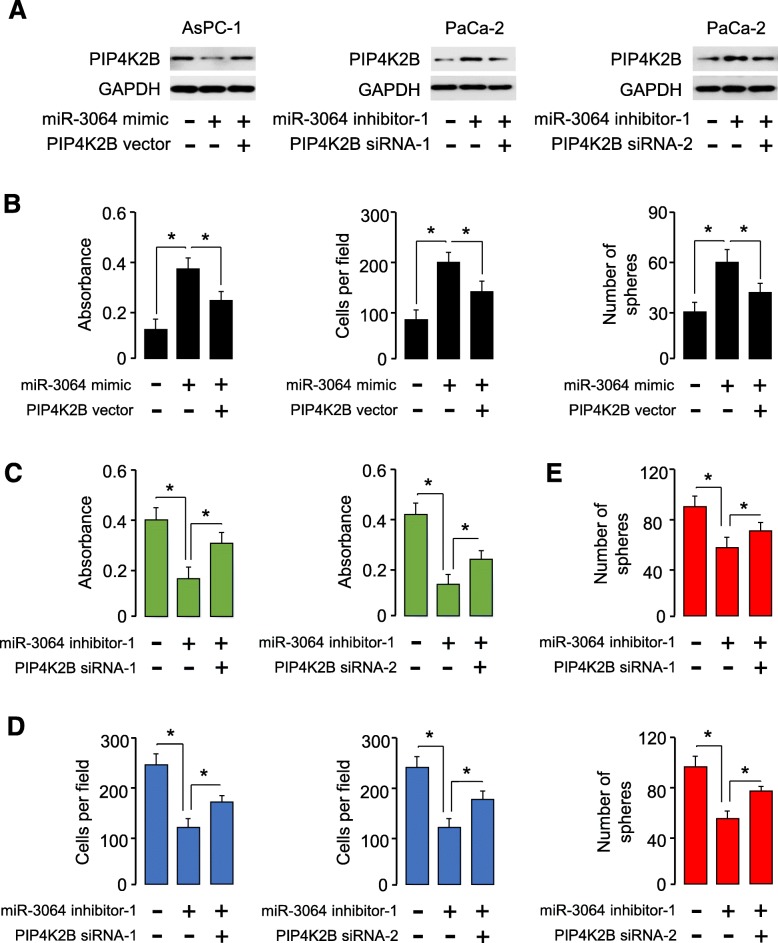
Restoration of PIP4K2B reverses the tumor-promoting effects of miR-3064 in PC cells. (**a**) PIP4K2B protein expression in AsPC-1 cells transfected with miR-3064 mimic with (or without) PIP4K2B expression vector, and in PaCa-2 cells transfected with miR-3064 inhibitor with (or without) PIP4K2B siRNAs. (**b**) Cell proliferation, invasion and sphere formation assays in AsPC-1 cells transfected with miR-3064 mimic with (or without) PIP4K2B expression vector. (**c**, **d**, **e**) Cell proliferation (**c**), invasion (**d**) and sphere formation assays (**e**) in PaCa-2 cells transfected with miR-3064 inhibitor with (or without) PIP4K2B siRNA-1 and siRNA-2. *P < 0.05

We tested whether the tumor-promoting effects of miR-3064 on PC cell proliferation, invasion and sphere formation were dependent on the expression of PIP4K2B. To do so, we restored PIP4K2B expression in AsPC-1 cells overexpressing miR-3064, and downregulated PIP4K2B levels in PaCa-2 cells with miR-3064 knockdown (Fig. 5a). We found that restoration of PIP4K2B expression in AsPC-1 cells was sufficient to reduce cell proliferation, invasion and sphere formation that was increased by miR-3064 overexpression (Fig. 5b), and knockdown of PIP4K2B expression significantly induced the proliferation, invasion and sphere formation of PaCa-2 cells that were repressed by miR-3064 knockdown (Fig. 5c and d). Therefore, we demonstrate that PIP4K2B acts as a tumor suppressor and a functional downstream effector of miR-3064 in PC cells.

### PXN-AS1 acts as a sponge for miR-3064 to repress its expression in PC cells

**Fig. 6:**
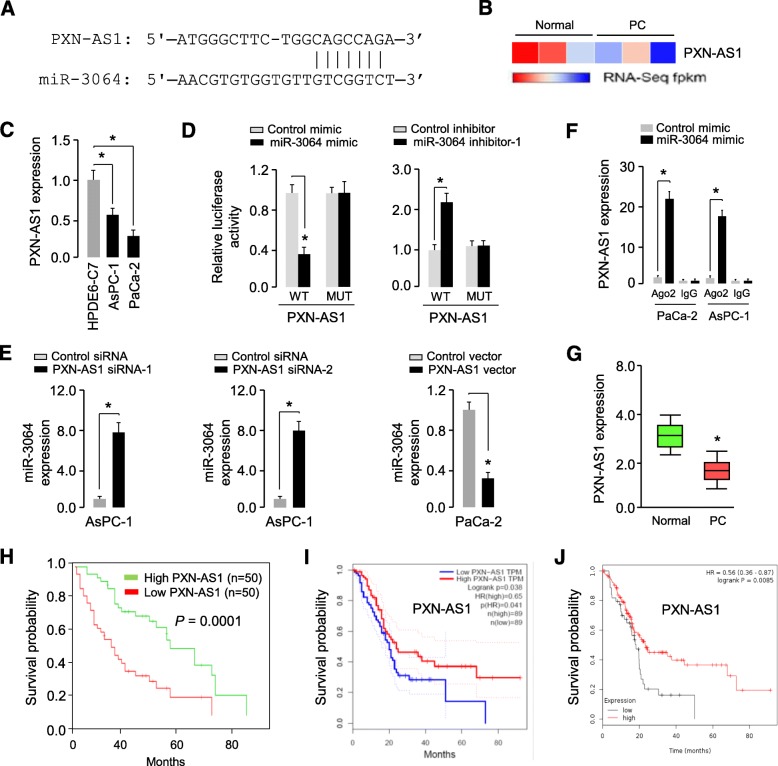
PXN-AS1 acts as a sponge for miR-3064 to repress its expression in PC cells. (**a**) Putative miR-3064 binding site in PXN-AS1 sequence. (**b**) The expression level of PXN-AS1 in PC tissues and normal tissues from the TCGA PC datasets (the lncRNAtor database). (**c**) The expression of PXN-AS1 was measured in PC cell lines and a normal pancreatic cell line HPDE6-C7 using qRT-PCR analysis. (**d**) Luciferase reporter assays were conducted to confirm the direct binding between miR-3064 and PXN-AS1. (**e**) qRT-PCR analysis of miR-3064 expression in PC cells following knockdown or overexpression of PXN-AS1. (**f**) RIP assays were performed in PC cells transfected with or without miR-3064 mimic. The expression of PXN-AS1 was examined using qRT-PCR analysis. (**g**) Analysis of PXN-AS1 expression in PC tissues and normal tissues using qRT-PCR assays. (**h**) Kaplan-Meier curves for overall survival of PC patients with low or high PXN-AS1 levels. (**i**) Kaplan-Meier curves for overall survival of PC patients with high or low levels of PXN-AS1 (the GEPIA database). (**j**) Kaplan-Meier curves for overall survival of PC patients with high or low levels of PXN-AS1 (the KMPlotter database). *P < 0.05

Using the online software starBase v2.0 [17], we found that lncRNA PXN-AS1 could form a complementary base pairing with miR-3064 (Fig. 6a). To investigate the role of PXN-AS1 in PC, we first evaluated the expression level of PXN-AS1 in PC tissues and normal tissues from the TCGA PC datasets using the lncRNAtor database (http://lncrnator.ewha.ac.kr/index.htm). The results showed that PXN-AS1 expression was significantly lower in PC tissues than that in normal tissues (Fig. 6b). Then, the expression of PXN-AS1 was measured in PC cell lines and a normal cell line HPDE6-C7 using qRT-PCR analysis. We observed that PXN-AS1 was significantly downregulated in PC cells (Fig. 6c), indicating that PXN-AS1 expression was negatively correlated with miR-3064 level in PC cells.

To confirm the direct binding between PXN-AS1 and miR-3064, the luciferase reporter assays were performed. The transfection with miR-3064 mimic resulted in an obvious suppression of luciferase activity of WT PXN-AS1, while the transfection with miR-3064 inhibitor led to a marked increase of luciferase activity of WT PXN-AS1 (Fig. 6d). In contrast, the introduction of miR-3064 mimic or inhibitor had no significant influence on the luciferase activity of MUT PXN-AS1 (Fig. 6d), suggesting that PXN-AS1 could directly interact with miR-3064. To investigate the impact of PXN-AS1 on the expression of miR-3064, we used qRT-PCR assays to show that the levels of miR-3064 was significantly induced in AsPC-1 cells with PXN-AS1 knockdown, but was significantly reduced in PaCa-2 cells overexpressing PXN-AS1 (Fig. 6e). To explore the associations between PXN-AS1 and miR-3064, RIP assay was performed on PC cell extracts using the Ago2 antibody. We found that PXN-AS1 was significantly enriched in the cells of miR-3064 mimic-transfected PC cells (Fig. 6f). These results show that PXN-AS1 sponges and suppress the expression of miR-3064 in PC cells.

To evaluate the clinical importance of PXN-AS1 in PC, we performed qRT-PCR analysis and found that PXN-AS1 level was downregulated in PC tissues compared with normal tissues (Fig. 6g). We verified that downregulation of PXN-AS1 was significantly correlated with increased tumor size, advanced TNM stage and the presence of lymph node metastasis in PC patients (Table 3).

**Table 3:** Correlations between PXN-AS1 expression and clinicopathological characteristics of PC patients

Characteristics	PXN-AS1 expression		*P*-value
	Low	High	
Age (years)			
> 60	11	16	0.615
≤ 60	11	12	
Differentiation			
Well/moderate	13	13	0.355
Poor	16	8	
Tumor size			
≤ 2 cm	3	15	**0.00007**
> 2 cm	24	8	
TNM Stage			
I/II	1	20	**0.00001**
III/IV	21	8	
Lymph node metastasis			
Positive	22	8	**0.0002**
Negative	4	16	

*Statistically significant (*P* < 0.05)

Notably, a significant association between high PXN-AS1 expression and longer overall survival was observed in our cohort (Fig. 6h). Furthermore, the Kaplan-Meier analysis using the GEPIA database (http://gepia.cancer-pku.cn/index.html) and KMPlotter database consistently suggested that the patients with higher PXN-AS1 expression exhibit better overall survival (Fig. 6i and j). Taken together, our data suggest that PXN-AS1 may retard the progression of PC by decreasing miR-3064 expression.

### PXN-AS1 suppresses PC cell proliferation, invasion and sphere formation partly through inhibiting miR-3064 expression

**Fig. 7:**
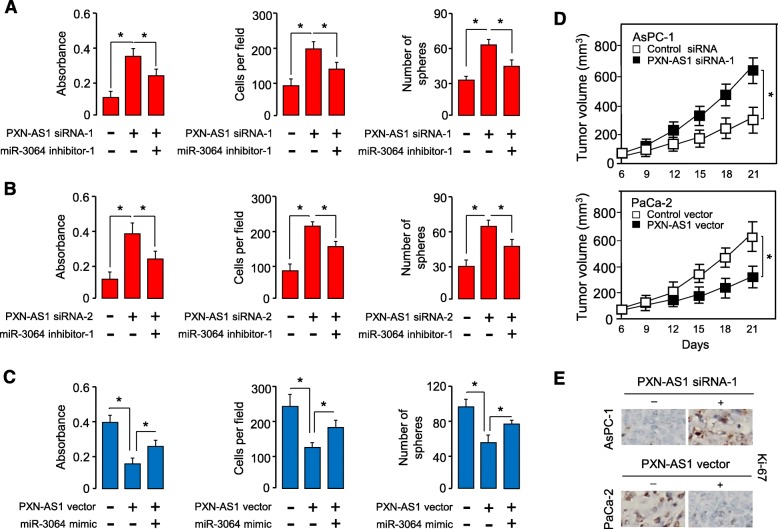
PXN-AS1 suppresses PC cell proliferation, invasion and sphere formation partly through inhibiting miR-3064 expression. (**a**, **b**) Cell proliferation, invasion and sphere formation assays in AsPC-1 cells transfected with PXN-AS1 siRNA-1 (**a**), or PXN-AS1 siRNA-2 (**b**), along with (or without) miR-3064 inhibitor. (**c**) Cell proliferation, invasion and sphere formation assays in PaCa-2 cells transfected with PXN-AS1 expression vector, along with (or without) miR-3064 mimic. (**d**) PC cells were transfected with PXN-AS1 siRNA-1 or PXN-AS1 expression vector as indicated, and then injected into nude mice. Tumor growth rates of xenograft tumor were shown. (**e**) Immunohistochemical staining of Ki-67 in tumors derived from (**d**). *P < 0.05

To clarify whether PXN-AS1 regulates PC cell growth, invasiveness and sphere formation via modulating the expression of miR-3064, we downregulated miR-3064 expression in AsPC-1 cells with PXN-AS1 knocked down and performed cell proliferation, invasion and sphere formation assays. Knockdown of PXN-AS1 significantly increased cell proliferation, invasion and sphere formation, the simultaneous depletion of miR-3064 partly reversed these effects (Fig. 7a and b). To further confirm the above results, we co-transfected PaCa-2 cells with the PXN-AS1 expression vector along with miR-3064 mimic, and observed that forced expression of miR-3064 could reverse the PXN-AS1 overexpression-mediated inhibition of cell proliferation, invasion and sphere formation (Fig. 7c).

**Fig. 8:**
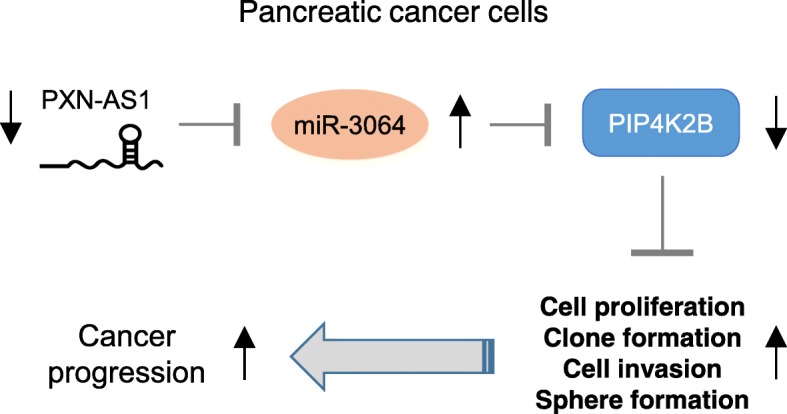
A schematic diagram depicting a potential mechanism by which the lncRNA PXN-AS1/miR-3064/PIP4K2B axis regulates the proliferation, invasion and sphere formation of PC cells

We subsequently assessed the effects of either overexpression or knockdown of PXN-AS1 on PC formation in vivo*.* Inhibition of PXN-AS1 significantly increased tumor growth (Fig. 7d). However, the tumors formed from the PXN-AS1-overexpressing PC cells showed slower growth rates than that of control cells (Fig. 7d). Immunostaining results showed that the knockdown of PXN-AS1 elevated the expression of Ki-67 (Fig. 7e). In contrast, the overexpression of PXN-AS1 exhibited the opposite effects (Fig. 7e). In summary, all these results collectively verified lncRNA PXN-AS1 as a key tumor suppressor that represses the proliferation, invasion and sphere formation of PC cells via downregulating the expression of oncogenic miR-3064 (Fig. 8).

## Discussion

PC is one of the worlds’ most aggressive malignancies, and the development of PC is a complicated process involved in the accumulation of genetic and epigenetic changes. Further studies of the mechanisms of PC tumorigenesis and progression are required to reduce the mortality caused by this cancer. Recently, the dysregulation of miRNAs was shown to play an essential part in regulating cancer progression. In this study, our in vitro and in vivo experiments have given both gain-of-function and loss-of-function evidence, showing for the first time that miR-3064 was highly overexpressed in PC tissues and PC Patients with high miR-3064 expression had worse survival, indicating that miR-3064 might serve as a prognostic marker in patients with PC.

A previous study indicated that miR-3064 was downregulated in ovarian cancer tissues and can inhibit cell proliferation, epithelial-mesenchymal transition and invasion in ovarian cancer cells [11]. However, studies about the functions and regulatory mechanisms of miR-3064 are currently very limited. Our study demonstrated that the overexpression of miR-3064 has oncogenic roles in promoting the proliferation, clone formation, invasion and sphere formation of PC cells in vitro and enhancing tumor growth in vivo. In contrast, CRISPR/Cas9-mediated knockout of miR-3064 resulted in opposite phenotypes. Thus, these results demonstrated that miR-3064 might either act as a tumor suppressor or as an oncogene in different human cancers and represent a potential therapeutic target in the treatment of PC.

For miRNA loss-of-function, the use of miRNA antisense inhibitor oligonucleotides, knockouts and miRNA sponges have been described [18]. Although antisense miRNA inhibitors are especially useful for short term experiments, the robustness, specificity, and stability of this strategy are not highly satisfied [19]. CRISPR/Cas9-based genome editing system can be used effectively to create mutations in the target loci, providing a novel platform to downregulate the expression of selected genes or miRNAs [14]. Here, we have shown that genetic ablation of miR-3064 by CRISPR/Cas9 system suppressed aggressive phenotypes of PC cells in vitro and in vivo. These results suggest that CRISPR/Cas9-mediated genome editing holds great therapeutic promise, although further studies are required to address the concerns regarding the safety and efficacy of CRISPR/Cas9 system in cancer treatment [20].

Furthermore, we explored the target genes of miR-3064, and found that miR-3064 bound to the 3′-UTR of a phosphoinositide kinase PIP4K2B [21], resulting in a marked decrease in PIP4K2B expression in PC cells. It has been reported that low PIP4K2B expression was correlated with increased distant metastasis and with worse prognosis in breast cancer [22], implying that PIP4K2B may be a tumor suppressive factor in breast cancer. The same study investigated the role of PIP4K2B in regulating the growth of breast cancer cells, and the knockdown of PIP4K2B induces EMT characteristics in breast cancer cells [22]. In our current study, the introduction of PIP4K2B reversed the tumor-promoting effects of miR-3064 in PC cells, suggesting that the gain of miR-3064 expression could result in the attenuation of tumor suppressor PIP4K2B, resulting in enhanced PC progression.

LncRNAs can downregulate the expression of tumor suppressor miRNAs in cancer cells by acting as competing miRNA sponges [5–7]. For example, by interacting with miRNAs, several lncRNAs (including GAS5, XIST, NORAD, and Linc00511) modulate PC progression by enhancing the proliferation, migration and invasive capacity and angiogenesis of PC cells as well as tumor growth in vivo [23–26]. In this study, we examined the associations between miR-3064 and potential lncRNAs. As a result, our luciferase assays together with RIP assays demonstrated that lncRNA PXN-AS1 was an upstream regulator of miR-3064, and PXN-AS1 functioned as a molecular sponge for miR-3064 and significantly reduced the growth, invasiveness and sphere formation of PC cells. We also reported that PXN-AS1 was downregulated in PC tissues and high PXN-AS1 expression was correlated with better survival in PC patients. To the best of our knowledge, our study was the first one addressing the expression level and biological effects of PXN-AS1 in PC. Our results revealed that the loss of PXN-AS1 expression could induce the expression of miR-3064, thereby promoting the proliferation, invasion and sphere formation of PC cells. It remains to be determined whether PXN-AS1 can target other miRNAs to influence PC progression.

## Conclusions

Our results highlighted an important role for the PXN-AS1/miR-3064/PIP4K2B axis in the regulation of PC progression and indicated that this pathway may have the potential for being used in diagnosis and treatment for PC.

## Additional file

Additional file 1: **Figure S1.** Verification of the effects of miR-3064 inhibition on the proliferation, clone formation, invasion and sphere formation in PaCa-2 cells. (A) Silencing of miR-3064 via anti-miR-3064 inhibitor-2 was verified using qRT-PCR analysis. (B-E) CCK-8 assay (B), clone formation assays (C), cell invasion assays (D) and sphere formation assays (E) in PaCa-2 cells after knockdown of miR-3064. *P < 0.05. (PDF 95 kb)

## Data Availability

The datasets used and/or analyzed during this study are available from the corresponding author on reasonable request.

## Abbreviations

gRNA: Guide RNA
lncRNA: Long non-coding RNAs
MiRNAs: Micrornas
PC: Pancreatic cancer
