# Correction to: Long non-coding RNA PXN-AS1 suppresses pancreatic cancer progression by acting as a competing endogenous RNA of miR-3064 to upregulate PIP4K2B expression

**DOI:** 10.1186/s13046-020-01573-3

**Published:** 2020-05-07

**Authors:** Jiayan Yan, Yunxi Jia, Han Chen, Wei Chen, Xiaoying Zhou

**Affiliations:** 1 Department of Biliary-Pancreatic Surgery, Renji Hospital, School of Medicine, Shanghai Jiaotong University, Shanghai, 200127 China; 2 Department of endoscopy of geriatric gastroenterology, First Affiliated Hospital of Nanjing Medical University, Nanjing, 210029 China; 3 Department of gastroenterology, First Affiliated Hospital of Nanjing Medical University, Nanjing, 210029 China

**Correction to: J Exp Clin Cancer Res**

**https://doi.org/10.1186/s13046-019-1379-5**

In the original publication of this manuscript [1], there are errors in Fig. 3. The authors declare that the identified errors do not change the results or conclusions of this paper.

The images of Fig. 3F (control group and miR-3064-KO-2 group) were mistakenly selected and used. The revised Fig. 3 is shown below.

**Fig. 3:**
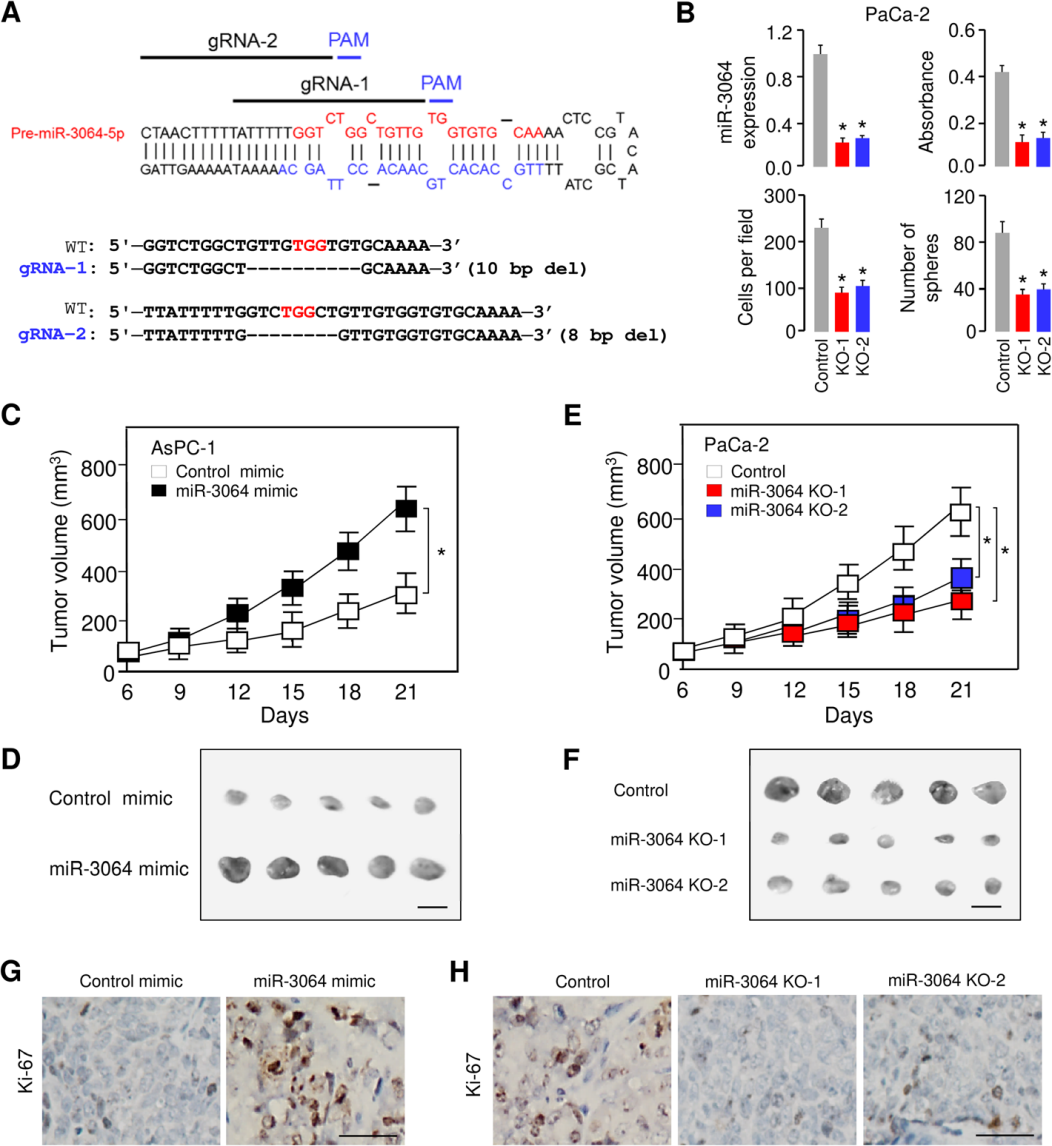
Depletion of miR-3064 suppresses the aggressive phenotypes of PC cells in vitro and inhibits tumor growth in xenograft mouse models. **a** Schematic diagram of gRNAs targeting at *miR-3064* locus (upper). DNA sequencing confirmed the deletions generated by CRISPR/Cas9 system in the *miR-3064* locus (bottom). **b** CRISPR/Cas9 with designed gRNAs significantly reduced the expression of miR-3064, and suppressed the proliferation, invasion and sphere formation of PaCa-2 cells. **c**, **d** AsPC-1 cells were transfected with or without miR-3064 mimic, and injected into nude mice. Tumor growth rates (**c**) and images (**d**) of xenograft tumors were shown. **e**, **f** The miR-3064 knockout or control PaCa-2 cells were injected into nude mice, and tumor growth rate (**e**) and images (**f**) of xenograft tumors were shown. **g**, **h** Immunohistochemical staining of Ki-67 in tumors derived from (**c** and **e**). *P < 0.05

In addition, scale bars are added into Figs. 3 and 7.

The authors apologize for the inconvenience caused to the readers.

**Fig. 7:**
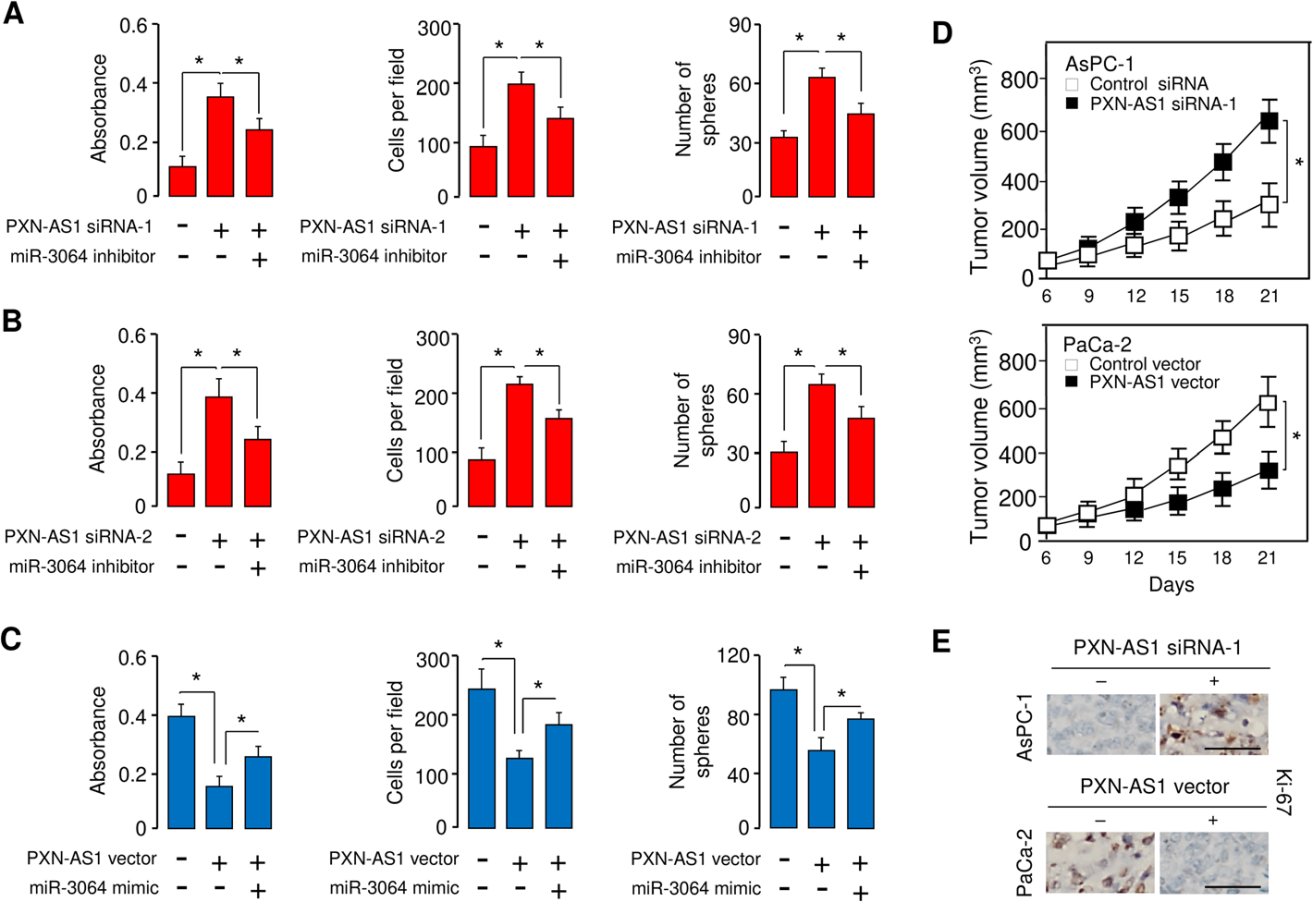
PXN-AS1 suppresses PC cell proliferation, invasion and sphere formation partly through inhibiting miR-3064 expression. (**a**, **b**) Cell proliferation, invasion and sphere formation assays in AsPC-1 cells transfected with PXN-AS1 siRNA-1 (**a**), or PXN-AS1 siRNA-2 (**b**), along with (or without) miR-3064 inhibitor. (**c**) Cell proliferation, invasion and sphere formation assays in PaCa-2 cells transfected with PXN-AS1 expression vector, along with (or without) miR-3064 mimic. (**d**) PC cells were transfected with PXN-AS1 siRNA-1 or PXN-AS1 expression vector as indicated, and then injected into nude mice. Tumor growth rates of xenograft tumor were shown. (**e**) Immunohistochemical staining of Ki-67 in tumors derived from (**d**). *P < 0.05
