# Supplementary figures and images for: Long non-coding RNA PXN-AS1 suppresses pancreatic cancer progression by acting as a competing endogenous RNA of miR-3064 to upregulate PIP4K2B expression
Source: J Exp Clin Cancer Res. 2019 Sep 5;38:390. doi: 10.1186/s13046-019-1379-5 (PMC6727519; doi:10.1186/s13046-019-1379-5)

Figure S1

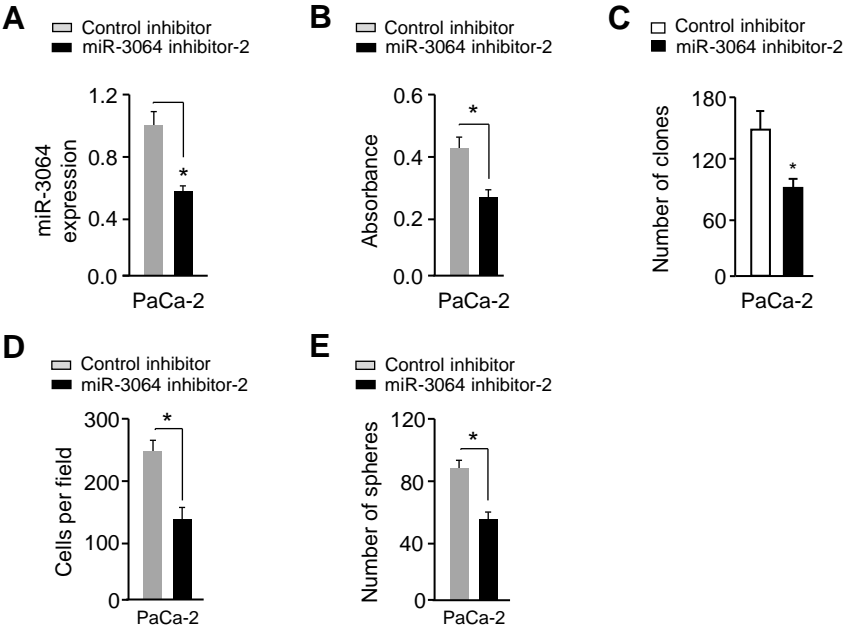

Supplement: Supplementary file 1 — Figure S1. Verification of the effects of miR-3064 inhibition on the proliferation, clone formation, invasion and sphere formation in PaCa-2 cells. (A) Silencing of miR-3064 via anti-miR-3064 inhibitor-2 was verified using qRT-PCR analysis. (B-E) CCK-8 assay (B), clone formation assays (C), cell invasion assays (D) and sphere formation assays (E) in PaCa-2 cells after knockdown of miR-3064. *P < 0.05. (PDF 95 kb) [file 13046_2019_1379_MOESM1_ESM.pdf]
